# CRISPR-Cas12a ribonucleoprotein-mediated gene editing in the plant pathogenic fungus *Magnaporthe oryzae*

**DOI:** 10.1016/j.xpro.2021.101072

**Published:** 2021-12-24

**Authors:** Jun Huang, David E. Cook

**Affiliations:** 1 Department of Plant Pathology, Kansas State University, Manhattan, KS, USA

**Keywords:** Genomics, Microbiology, Model Organisms, Plant sciences, Molecular Biology, CRISPR, Biotechnology and bioengineering

## Abstract

Gene replacements through homologous recombination (HR) have been extensively used for functional genomic studies. However, the general efficiency of HR repair can be low in filamentous fungi and the process laborious. Here, we provide a detailed protocol for efficient gene editing by inserting donor DNA into a region of interest following Cas12a ribonucleoprotein (RNP)-mediated DNA double-strand break. We demonstrate this protocol using *Magnaporthe oryzae* (synonym of *Pyricularia oryzae*), a model plant pathogenic fungus that is used to study plant-fungal interactions.

For complete details on the use and execution of this protocol, please refer to Huang et al. (2021).

## Highlights

• Protocol for designing and performing Cas12a genome editing in a filamentous fungus
• Donor DNA integration provides efficient transformation and selection
• The donor DNA does not require homologous sequence to the targeted locus
• The transformation and genotyping approach identifies many types of DNA mutations

## Graphical abstract

**Figure:**
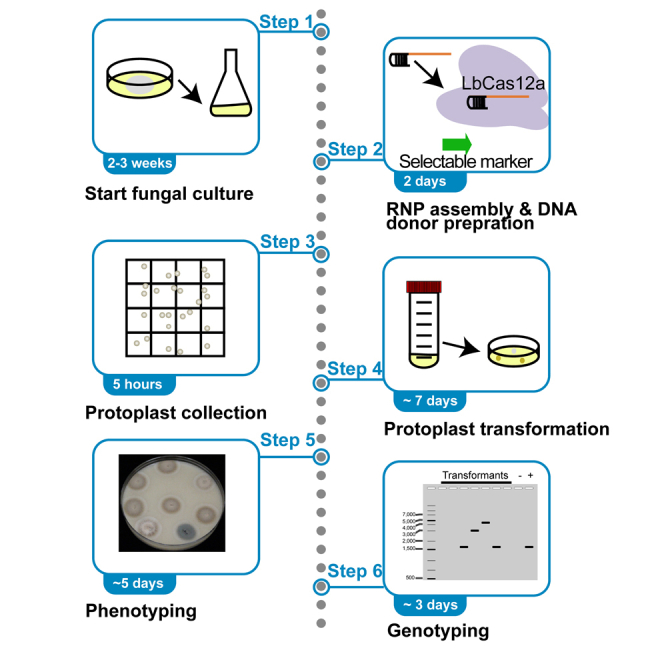

## Before you begin

**CRITICAL:** To avoid contamination, all the steps involving fungal protoplasts and cultures require a sterile work environment, such as a biosafety cabinet. This includes using sterilized materials and tools.

### Fungal culture

**Timing: 1–3 weeks**

1. Begin your starter culture on plates from a stock. For *M. oryzae*, transfer isolate from filter paper stock to oatmeal agar (OTA) using a sterilized toothpick.
2. Incubate the culture in a growth chamber at 25°C under light for 1–2 weeks.
3. Cut 5–8 small mycelial plugs (1–2 mm side length) from the OTA plate with a sterilized toothpick. Move the mycelial plugs into 100 mL liquid complete medium (CM) and grow at 28°C, 120 rpm for approximately 2–4 days in the dark.

**CRITICAL:** Check the fungal status daily to avoid melanized mycelia (i.e., mycelium turning black), which negatively affects the efficiency to obtain fungal protoplast.

## Key resources table

**Table:** 

REAGENT or RESOURCE	SOURCE	IDENTIFIER
**Chemicals, peptides, and recombinant proteins**		

Oatmeal Agar	BD	Cat#DF0552-17-3
Sucrose	Fisher Scientific	Cat#S5-12
Yeast extract	BD	Cat#21750
Casamino acid	VWR	Cat#J851-500G
Yeast Nitrogen Base Without Amino Acids and Ammonium Sulfate	Fisher Scientific	Cat#DF0335-15-9
Ammonium nitrate	Sigma-Aldrich	Cat#221244-500G
L- (+) - Asparagine	Fisher Scientific	Cat#AAB2147322
Glucose	Sigma-Aldrich	Cat#G8270-100G
Agarose, low gelling temperature	Sigma-Aldrich	Cat#A9414-5G
di-sodium hydrogen phosphate	Sigma-Aldrich	Cat#1065851000
Bacteriological Agar	IBI Scientific	Cat#IB49171
Hygromycin B in solution	Corning	Cat#45000-806
G418 solution	VWR	Cat#97064-358
Bialaphos/glufosinate ammonium	Crescent Chemical	Cat#C140300
FK506	LC Laboratories	Cat#NC0876958
Agarose	VWR	Cat#0710-500G
LbCas12a protein	New England BioLabs	Cat#M0653T
Lysing Enzymes from *Trichoderma harzianum*	Sigma-Aldrich	Cat#L1412-10G
Sodium chloride	Fisher Scientific	Cat#S271-3
PEG4000	FLUKA	Cat#81240
Potassium chloride	Sigma-Aldrich	Cat#P-4504
Chloroform	Sigma-Aldrich	Cat#C2432-1L
Isopropanol	Fisher Scientific	Cat#A416-4
Ethanol	Fisher Scientific	Cat#BP2818-500
Tris-HCI, pH 8.0	Thermo Fisher Scientific	Cat#AM9856
Calcium chloride	Sigma-Aldrich	Cat#C8106-1KG
GeneRuler 1 kb Plus DNA Ladder	Thermo Fisher Scientific	Cat#SM1331

**Critical commercial assays**		

Phusion® High-Fidelity DNA Polymerase	New England BioLabs	Cat#M0530L
Q5® High-Fidelity DNA Polymerases	New England BioLabs	Cat#M0491L
Taq DNA Polymerase with Standard Taq Buffer	New England BioLabs	Cat#M0273L
HiScribe™ T7 High Yield RNA Synthesis Kit	New England BioLabs	Cat#E2040S
Monarch® RNA Cleanup Kit	New England BioLabs	Cat#T2050L
DNase I (RNase-free)	New England BioLabs	Cat#M0303S
Deoxynucleotide (dNTP) Solution Mix	New England BioLabs	Cat#N0447L
Wizard SV Gel and PCR Clean-Up system	Promega	Cat#A9282

**Experimental models:Organisms/strains**		

*M. oryzae* field isolate O-137	Dr. Barbara Valent (Kansas State Univ)	N/A

**Oligonucleotides**		

Please see the oligos used in this protocol in Table S1	Huang et al. (2021)	N/A

**Recombinant DNA**		

pFGL821	Addgene	Cat#58223
pFGL921	Zhang et al. (2021)	N/A
pBV9/pSM324	Kankanala et al. (2007)	N/A

**Software and algorithms**		

BLAST	FungiDB	https://fungidb.org/fungidb/app/search/transcript/UnifiedBlast

**Other**		

Membrane Filter, 0.22 μm pore size	Millex	Cat#SLGVM33RS
Plastic syringes	Thermo Fisher Scientific	Cat#S7510-10
Toothpick	Fisher Scientific	Cat#S24554
Hemocytometer	Fisher Scientific	Cat#0267151B
Miracloth	Calbiochem	Cat#475855-1R
6_1/2_ in Disks Non Gauze Milk Filter	KenAG	N/A
Water, Molecular Biology Grade	VWR	Cat#VWRL0201-0500
150 × 15 mm petri dishes	Fisher Scientific	Cat#FB0875714
60 × 15 mm Petri dishes	MIDSCI	Cat#901
100 × 15 mm Petri dishes	MIDSCI	Cat#900
50 mL falcon tubes	MIDSCI	Cat#C50B
1.5 mL microcentrifuge tubes	USA scientific	Cat#1615-5500
0.2 mL PCR tubes	MIDSCI	Cat#AVTW-F
Funnels	Fisher Scientific	Cat#FB6015865
125 mL flask	PYREX	N/A
200 mL flask	PYREX	N/A
Parafilm	Sigma-Aldrich	Cat#P7793-1EA
NanoDrop spectrophotometer	Thermo Fisher Scientific	N/A
2.0 mL impact resistant screw cap tube	USA scientific	Cat #1420-9600
1.0 mm Silica Beads	BioSpec	Cat #11079110z
Bead ruptor elite-Bead mill homogenizer	OMNI international	Cat#19-2141E
Centrifuge	Eppendorf	Cat#5415D
Centrifuge	Beckman Coulter	Cat#Allegra 25R
Filter paper	N/A	N/A

## Materials and equipment

### Prepare medium and stock solution

**Timing: 1 week**

#### Oatmeal agar (OTA)

Dissolve 72.5 g commercial OTA powder in 1 L of distilled water, mix well. Heat to near boiling using a microwave for 1–2 min. Autoclave at 121°C for 20 min in liquid cycle. Pour the sterilized OTA in a 100 × 15 mm petri dish after autoclave, store at room temperature (∼28°C). The plate can be stored for up to 2 months.

**CRITICAL:** It is very important to preheat the OTA before autoclave steps.

***Alternatives:*** Microwave can be replaced with a water bath for pre-heating.

**Table:** Complete medium (CM)

Reagent	Final concentration	Amount
Sucrose	10 g/L	10 g
Yeast extract	6 g/L	6 g
Casamino acid	6 g/L	6 g
Agar	20 g/L	20 g
ddH_2_O	n/a	up to 1000 mL
**Total**	**n/a**	**1000 mL**

Autoclave at 121°C for 20 min in liquid cycle. Agar is not required for liquid CM.

Store at room temperature up to 6 months.

**Table:** TB3 medium (TB3)

Reagent	Final concentration	Amount
Sucrose	200 g/L	200 g
Yeast extract	6 g/L	6 g
Casamino acid	6 g/L	6 g
Agar	15 g/L	15 g
ddH_2_O	n/a	up to 1000 mL
**Total**	**n/a**	**1000 mL**

Autoclave at 121°C for 20 min in liquid cycle. Agar is not required for liquid TB3.

Store at room temperature up to 6 months.

**Table:** Defined complex medium (DCM)

Reagent	Final concentration	Amount
Yeast nitrogen base without amino acids and ammonium sulphate	1.7 g/L	1.7 g
Ammonium nitrate	1 g/L	1 g
Asparagine	2 g/L	2 g
Glucose	10 g/L	10 g
Agarose, low gelling temperature	20 g/L	20 g
ddH_2_O	n/a	up to 1000 mL
**Total**	**n/a**	**1000 mL**

Adjust pH to 6 with di-sodium hydrogen phosphate (Na_2_HPO_4_). Autoclave at 121°C for 15 min in liquid cycle. Store at room temperature up to 6 months.

**Table:** 1×STC solution

Reagent	Final concentration	Amount
Sucrose	20% w/v	80 g
1M Tris-Hcl pH = 8.0	50 mM	20 mL
CaCl_2_. 2H_2_O	50 mM	2.94 g
ddH_2_O	n/a	up to 400 mL
**Total**	**n/a**	**400 mL**

Autoclave at 121°C for 20 min in liquid cycle. Store at 4°C for up to 6 months

**Table:** PTC solution

Reagent	Final concentration	Amount
PEG4000	60% w/v	60 g
1× STC solution	n/a	up to 100 mL
**Total**	**n/a**	**100 mL**

Autoclave at 121°C for 20 min in liquid cycle. Store at room temperature for up to 6 months.

If the solution is not completely dissolved before you use, microwave PTC solution to help it dissolve.

#### 0.7 M NaCl solution

Dissolve 16.36 g NaCl in 400mL of distilled water. Autoclave at 121°C for 20 min in liquid cycle. Store at room temperature for up to 6 months.

**Table:** Protoplast lysing solution

Reagent	Final concentration	Amount
Lysing Enzymes from *Trichoderma harzianum*	10 mg/mL	0.3 g
0.7M NaCl solution	n/a	up to 30 mL
**Total**	**n/a**	**30 mL**

Filter sterilize using 0.22 μm filter. This solution needs to be freshly prepared on the date when you are ready to perform protoplast preparation.

#### 1 M KCl solution

Dissolve 29.8 g KCl in 400 mL of distilled water. Autoclave at 121°C for 20 min in liquid cycle. Store at room temperature for up to 6 months.

#### Vector usage

We amplified the coding sequence for hygromycin, g418 and Bialaphos from the vectors pFGL821, pFGL921 and pBV9/pSM324 respectively. Additional details can be found in the Note of step 9. Other vectors or templates can additionally be used to amplify other genes of interest.

## Step-by-step method details

### Design oligos for LbCas12a guide RNA

**Timing: 1 week**

This step is required to design a guide RNA that will direct the Cas12a nuclease to a specific site for editing. To demonstrate CRISPR-Cas12a editing in *M. oryzae*, we mutated the trihydroxynaphthalene reductase encoding gene termed *BUF1* (MGG_02252), which is involved in fungal melanin biosynthesis (Chumley and Valent, 1990). The *buf1* deletion mutant showed buff color (i.e., tan/orange) compared with the gray/black mycelial color in the wild-type strain. In this step, we describe the procedure to design the Cas12a guide RNA targeting *BUF1*.

**Figure 1:**
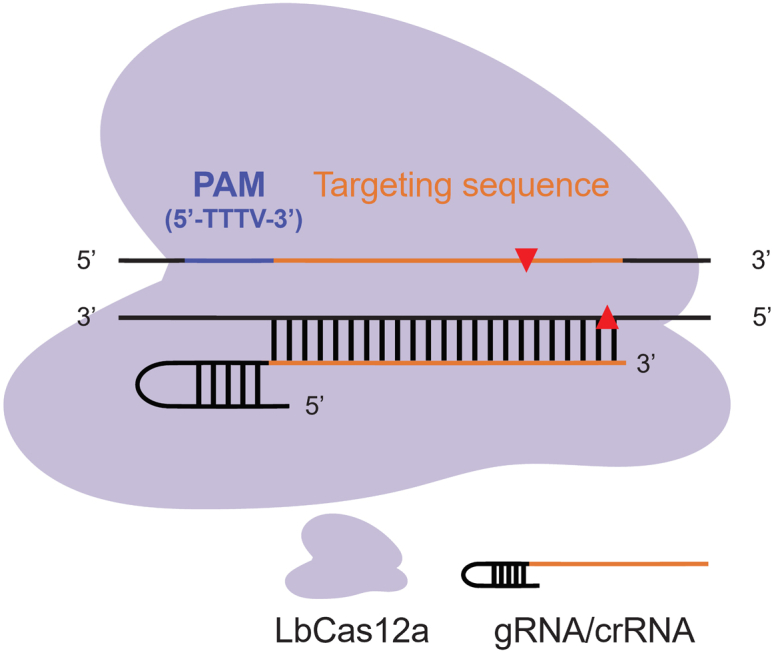
Schematic diagram of Cas12a cutting Blue line highlights the PAM sequence, the orange line indicates the designed targeting sequence. Arrows indicate the cutting site (17th base after PAM in the non-targeted strand and 22nd base after PAM in the targeted strand) of Cas12a, which is represented by the purple shape. The gRNA contains a hairpin at the 5′ end to facilitate Cas12a interaction.

1. Download the sequence of gene of interest from FungiDB (https://fungidb.org/fungidb/app). For the *BUF1* gene, download the sequence for the gene MGG_02252.
2. Search for the LbCas12a PAM sequences (5′-TTTV-3′) in your gene of interest. Select a 23 bp DNA sequence downstream (3′ end) of PAM as a targeting sequence (Figure 1). In the case of *BUF1*, we designed two spacer/targeting sequences.

*BUF1*-targeting sequence#1: **TTTC**TCACCTTCAGCTCACTTCCCCAA

*BUF1*-targeting sequence#2: **TTTA**CCATCAACACCCGTGGCCAGTTC

First four **bold** letters are the PAM, the remaining 23 bp are the targeting sequences.

***Note:*** For LbCas12a guide designing, the targeting sequences must be on the same strand of PAM (‘5-TTTV-3’). V indicates A, C or G, but not T. Previous research reported that targeting sequences with GC-content (∼40%–60%) provides the highest editing activity for Cas12a (Kim et al., 2017). We advise avoiding targeting sequences with GC content <30% or >70% (Kim et al., 2017).

3. Check the off-target effect by blasting the designed targeting sequences within FungiDB database (https://fungidb.org/fungidb/app/search/transcript/UnifiedBlast)
  a. Choose ‘genome’ as Target Data Type
  b. Choose ‘blastn’ as BLAST Program
  c. Choose ‘*Pyricularia oryzae* 70-15’ as Target organism
  d. Input 27 bp sequences (4 bp PAM + 23 bp targeting sequences)
  e. If the blast result shows the other loci match first 4 bp PAM and at least 16 bp targeting sequences after PAM perfectly, it may cause off-target editing. It is best to discard this potential guide sequence. Otherwise, move forward with the targeting sequence design.

***Note:****Pyricularia oryzae* is a synonym of *Magnaporthe oryzae*.

**Figure 2:**
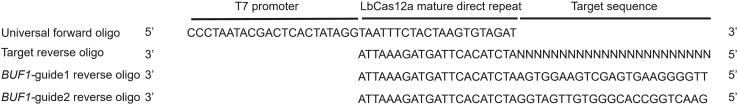
Schematic examples of oligo design
  b. Target specific reverse oligo including the reverse complemented sequences of LbCas12a mature direct repeat and the 23 bp targeting sequences you designed for your gene of interest (Figure 2). Oligos designed for two guides targeting the *BUF1* locus, termed *BUF1*-guide1 and *BUF1*-guide2, are provided as examples (Figure 2).

***Note:*** Do not include PAM sequence in your target reverse oligo.

***Note:*** Various online tools have been developed for genome-wide CRISPR target design. Many are dependent on the organism of interest and must be checked for suitability.

4. Design oligos for preparing the DNA template for *in vitro* guide RNA synthesis. Two oligos are designed to construct the DNA template.
  a. Universal forward oligo including the T7 promoter and LbCas12a mature direct repeat (Figure 2).

***Optional:*** Commercial guide RNA synthesis kits, compatible with different Cas proteins, are available as an alternative option for preparing gRNA. For example, the NEB EnGen® sgRNA Synthesis Kit for the *S. pyogenes* SpCas9 protein can be used for making the corresponding gRNA (NEB, Cat#E3322V).

**CRITICAL:** If high-quality genome assembly of your isolate is not available, you can check the potential off-target effects of your guides with the commonly used 70-15 genome assembly (Dean et al., 2005).

### *In vitro* guide RNA synthesis and RNP assembly

**Timing: 2 days**

Following guide RNA design, this step is required to synthesize and associate the guide RNA with the Cas12a protein. This results in a competent RNP ready for transformation.

5. Generate DNA template for *in vitro* guide RNA synthesis. Set up the below PCR reaction with Phusion® High-Fidelity DNA Polymerase.

**Table:** 

Reagent	Amount
5× Phusion HF Buffer	4 μL
10 mM dNTPs	0.8 μL
Universal forward primer (100 μM)	0.4 μL
Target specific reverse primer (100 μM)	0.4 μL
Phusion DNA Polymerase	0.2 μL
Molecular water	14.2 μL
**Total**	**20 μL**

**Table:** PCR cycling conditions

Steps	Temperature	Time	Cycles
Initial Denaturation	95°C	30 s	1
Denaturation	95°C	10 s	35 cycles
Annealing	57°C	10 s	
Extension	72°C	10 s	
Final extension	72°C	2 min	1
Hold	4°C	2 min

**CRITICAL:** Make sure to use the correct concentration of oligos in the reaction. Products from this step do not require purification. The quality of the amplification reaction can be checked by running 2 μL of PCR product on a 2% agarose gel and visualization. The product should be 66 bp.

***Optional:*** Ordering the top and bottom oligos with full sequences (i.e., T7 promoter, LbCas12a mature direct repeat and 23 bp targeting sequences), and annealing them as the DNA template would serve as an alternative option to avoid this PCR step.

6. Guide RNA *in vitro* transcription. Set up the transcription as below by using HiScribe™ T7 High Yield RNA Synthesis Kit (NEB, Cat#E2040S).

**Table:** 

Reagent	Amount
10× Reaction Buffer	2 μL
ATP (100 mM)	2 μL
GTP (100 mM)	2 μL
CTP (100 mM)	2 μL
UTP (100 mM)	2 μL
Prepared template DNA from step 5	8 μL
T7 RNA Polymerase Mix	2 μL
**Total**	**20 μL**

Run the reaction in a thermocycler with 37°C for 8–18 h.

***Note:*** Based on our experience, the yield should be at least 50 μg RNA from this step.

7. Purification of synthetic guide RNA
  a. Remove the DNA template by adding 1 μL (2 units) RNase-free DNase to the guide RNA reaction and incubate for 15 min at 37°C in a thermocycler.
  b. Purify the DNase treated guide RNA through Monarch® RNA Cleanup Kit (NEB Cat#T2050L) followed the manufacturer's protocol (https://www.neb.com/protocols/2018/06/28/monarch-rna-cleanup-kit-protocol).
  c. Quantify the RNA quality by NanoDrop spectrophotometer. A 260/280 ratio of 2.0–2.2 following guide RNA transcription and purification indicates successful synthesis.
  d. Use guide RNA immediately for RNP assembly or store at −80°C for up to 2 months.
8. Assemble the RNP for one reaction in a 0.2 mL PCR tube as indicated below.

**Table:** 

Reagent	Final concentration	Amount
LbCas12a protein (100 μM)	∼ 5 ug/20 μL	0.34 μL
gRNA	∼ 0.5 ug/20 μL	x μl
NEBuffer 2.1 Reaction Buffer (10×)	n/a	2 μL
Molecular water	n/a	up to (17.66-x) μl
**Total**	**n/a**	**20 μL**

The amount of gRNA depends on the concentration of gRNA (e.g., if the concentration is 1000 ng/μL, then use x = 0.5 μL for the reaction).

Incubate the above reaction for 15 min at 25°C using a thermocycler.

***Note:*** Add the LbCas12a protein last to avoid protein precipitation (Fernandez et al., 2018). Assemble the RNP immediately before fungal protoplast transformation.

### Donor DNA preparation

**Timing: 5–6 h**

This protocol uses donor DNA for selection, which is integrated at the Cas12a DNA double-strand break site. This step is used to amplify and purify the donor DNA prior to transformation.

9. Amplify the no-homology *HYG* DNA donor as indicated below

**Table:** 

Reagent	Amount
5× Phusion HF Buffer	10 μL
10 mM dNTPs	1 μL
no-homology *HYG* DNA forward primer (10 μM)	2.5 μL
no-homology *HYG* DNA reverse primer (10 μM)	2.5 μL
Phusion DNA Polymerase	0.5 μL
pFGL821 (∼5 ng/μL)	1 μL
Molecular water	32.5 μL
**Total**	**50 μL**

**Table:** PCR cycling conditions

Steps	Temperature	Time	Cycles
Initial Denaturation	98°C	30 s	1
Denaturation	98°C	10 s	30 cycles
Annealing	57°C	30 s	
Extension	72°C	1 min	
Final extension	72°C	10 min	1
Hold	4°C	2min

***Note:*** Expected PCR amplification size is 1,466 bp. Each fungal protoplast transformation requires ∼3 μg donor DNA in total. Adjust the number of above PCR reactions based on the total number of transformations you will run. We amplified the *HYG* DNA donor by using pFGL821 plasmid as DNA template. Other selectable markers (e.g., G418 and Bialaphos) could be used. We amplified G418 coding sequence from plasmid pFGL921 and Bialaphos coding DNA from plasmid pBV9/pSM324 (Kankanala et al., 2007; Zhang et al., 2021). If the gene being edited can be directly used for selection, such as editing *FKBP12* (Huang et al., 2021) or *URA3*/*URA5* as has been shown in *Fusarium oxysporum* for instance (Wang et al., 2018), the inclusion of donor DNA in the experiment is not necessary.

***Note:*** Selection based on *URA3*/*URA5* has not been established in *M. oryzae*, and preliminary experiments might be needed to confirm selection based on *URA3*/*URA5* in *M. oryzae*.

10. Check the amplification quality by running 2 μL of PCR product on a 1% agarose gel. Successful amplification should yield a single product of the expected size.
11. Purify the PCR reaction through Wizard SV Gel and PCR Clean-Up system (Promega). The detailed purification step follows manufacturer's protocols (https://www.promega.com/-/media/files/resources/protocols/technical-bulletins/101/wizard-sv-gel-and-pcr-clean-up-system-protocol.pdf?la=en). If there are more products from the amplification product as indicated by step 10, gel purification can be used to isolate the product of interest by using the same Wizard SV Gel and PCR Clean-Up system (Promega).
12. Quantify the DNA quality by NanoDrop spectrophotometer. Donor concentration near 200 ng/μL–350 ng/μL is preferred.

***Note:*** Store the DNA donor in −20°C freezer until future use.

### *M. oryzae* protoplast preparation

**Timing: 5–6 h**

This step is required to generate the necessary *M. oryzae* cells to be used for transformation. The protoplasts are obtained by degrading the fungal cell wall, and allows more efficient RNP and nucleic acid transfer.

13. Prepare the protoplast lysing solution freshly as described in “prepare medium and stock solution” section.
14. Collect the mycelia from sub-cultured liquid CM through 2-layers of 6_1/2_ in Disks Non Gauze Milk Filter papers and funnel. Wash the collected mycelia with a 5 mL 1×STC solution.
15. Dry the collected mycelia with extra filter paper, transfer the dry mycelia to 100 mL flask with 25–30 mL protoplast lysing solution. Shake dry mycelia containing the flask at 30°C, 70–80 rpm, for 2.5–3 h under dark to digest the fungal cell wall and release protoplasts.

***Note:*** When transferring the mycelium, manually separate the mass so it is not a single large mass. This will increase the accessibility to the lysing solution.

**Figure 3:**
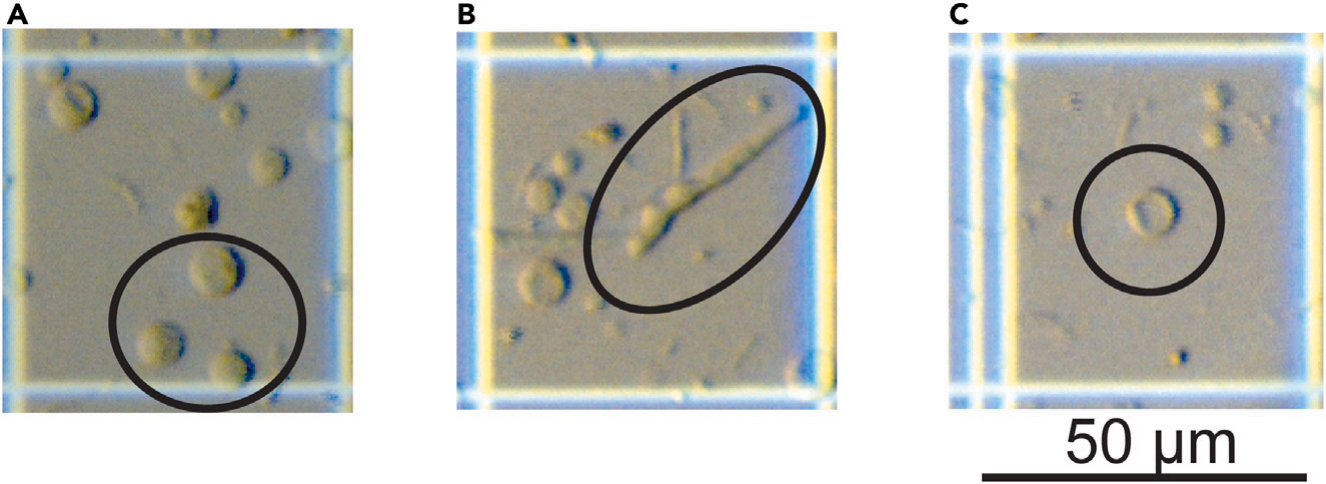
Different protoplast status after lysing enzyme digestion (A–C) (A) An example of healthy protoplasts, (B) undigested mycelium, and (C) collapsed protoplasts each highlighted by black circles. Scale Bars = 50 μm
17. Collect the protoplast by filtering the above lysing reaction containing the fungal protoplasts, through 2-layer miracloth into pre-chilled, 50 mL falcon tube. Wash the lysing debris left in the miracloth with 1×STC again to collect all the protoplasts.
18. Centrifuge the 50mL falcon tube with protoplast at 4°C, 5,000 rpm (i.e., 3,829 g in Beckman Coulter-Allegra 25R centrifuge) for 10 min.

***Note:*** A visible pellet containing protoplasts should be seen.

16. Check the digestion progress and protoplast quality using a hemocytometer under a dissecting microscope. Successful digestion should release many healthy protoplasts, while if many undigested mycelia are observed, extend the digestion time. However, excessive digestion can also cause protoplast collapse (Figure 3).

19. Discard the supernatant into a biohazard trash container. Gently resuspend the protoplast using 5–10 mL 1×STC by pipetting or flicking.
20. Repeat step 18.
21. Discard the supernatant into a biohazard trash container, and gently resuspend the protoplast in 1mL 1×STC. Quantify the protoplast concentration using a hemocytometer. Add additional 1×STC to adjust the final concentration of protoplasts to 8 × 10^6^ to 5 × 10^7^ protoplasts/mL. Aliquot 200–250 μL protoplast solution to individual 50 mL falcon tubes for further transformation.

***Optional:*** The protoplasts can be frozen and stored by adding 7% v/v DMSO into the protoplast solution and freezing at −80°C. We recommend performing the protoplast transformation immediately after preparation for best results, but the frozen cultures can still be used for successful editing.

***Note:*** This protocol is strain independent, and we have used it to make protoplasts for various isolates including O-137 and Guy11 (Bao et al., 2017).

### *M. oryzae* protoplast transformation

**Timing: 2 days**

This step transfers the assembled RNP and donor DNA into the fungal cell to allow editing and donor DNA integration.

22. Slowly add 20 μL assembled RNP and 3 μg of no-homology *HYG* DNA donor into the 50 mL falcon tube containing the aliquoted protoplast. Mix the solution by flicking gently. Place the tube at room temperature for 20–25 min.

***Note:*** If your gene of interest can be used as selection directly, DNA donor is not required in step 22 For example, we have generated *fkbp12* (FK506 receptor) mutants by using the selection of drug FK506 without DNA donor successfully (Huang et al., 2021).

23. Slowly add 1,000 μL PTC solution to the 50 mL falcon tube. Mix the solution by flicking gently. Place the tube at room temperature for 20–25 min.
24. Slowly add 5 mL liquid TB3 medium into the 50 mL falcon tube. Mix the solution by flicking gently. Cover the 50 mL falcon tube lid with parafilm.
25. Shake 50 mL falcon tube at 28°C, 90 rpm overnight (12–20 h) in the dark.
26. Next day, unseal the 50 mL falcon tube and add melting TB3 medium (near 40 mL for one transformation, 50°C–60°C) with final concentration of 100 μg/mL hygromycin solution. Mix gently, then pour the mixture (overnight protoplast culture + medium) into 150 × 15 mm petri-dish. Wait for about 20 min until complete solidification.

***Note:*** Protoplasts are fragile and require gentle handling. Other antibiotics corresponding to a different no-homology DNA donor can also be used. For example, we have also used G418 at a final concentration: 300 μg/mL. Bialaphos also can be used at a final concentration: 25 μg/mL in DCM instead of TB3 medium.

27. After the first selection layer has completely solidified, pour a second layer of melting TB3 medium (near 50 mL) with final concentration of 200 μg/mL hygromycin solution into the 150 × 15 mm petri-dish above the first layer. Wait for about 20 min until complete solidification.

***Note:*** For G418 selection, the final concentration for the second layer is 600 μg/mL. Bialaphos also can be used at a final concentration: 100 μg/mL in DCM instead of TB3 medium.

28. Seal the 150 × 15 mm petri-dish with parafilm and incubate the plate upside down at 28°C for 5–7 days under dark.

***Note:*** Transformants using *M. oryzae* isolate O-137 can typically be seen and transferred at 5 days, while those from isolate Guy11 typically take 7 days. Other isolates will have to be empirically determined.

29. Pick the transformants growing in the top layer for further phenotyping and genotyping. This can be done using a sterile toothpick to move a piece of mycelium to a new plate containing the selection.
30. For *BUF1* deletion identification, we cultured the transformants to OTA to check the mycelial color change. For other genes of interest, transformants can be moved to CM with corresponding appropriate selection: 200 μg/mL hygromycin or 500 μg/mL G418. If you use bialaphos selection (final concentration: 100 μg/mL), select DCM instead of CM for screening.

### Quick fungal DNA extraction for genotyping

**Timing: 5–6 h**

DNA is extracted from transformants in order to genotype putative DNA edits.

31. Prepare the screw cap tubes with 1.0 mm beads, 400 μL 1M KCL and 400 μL chloroform (added in hood).
32. Add fungal plug (near 1 cm diameter) into the above prepared tubes. (Performed in the biosafety cabinet hood)
33. Put the above tubes in a homogenizer with power 4 × 6 cycles (30 s each cycle) to disrupt the fungal material.
34. Centrifuge 13,200 rpm (i.e.,16,100 *g* in Eppendorf 5415D centrifuge) × 10 min
35. Transfer 200 μL supernatant into new labeled tubes
36. Add 120 μL isopropanol in the tube, mix well to precipitate the DNA for at least 10 min (added in hood).
37. Centrifuge 13,200 rpm (i.e.,16,100 g in Eppendorf 5415D centrifuge) × 10 min.
38. Discard the supernatant and wash the pellet with 500 μL 75% ethanol.
39. Centrifuge 13,200 rpm (i.e.,16,100 g in Eppendorf 5415D centrifuge) × 10 min.
40. Discard the ethanol and air dry for 10 min.
41. Add 50 μL sterilized water to dissolve the DNA pellet.

***Note:*** We prefer to use 1 μL of the dissolved DNA as template for PCR amplification. The DNA extracted by this quick extraction protocol is sufficient for PCR genotyping but should not be considered adequate for other uses such as next-generation sequencing or southern blot.

### PCR genotyping

Using the extracted DNA, PCR is used to determine if the gene of interest has been mutated by amplifying multiple regions at the target locus. This allows the researcher to understand and identify a range of possible DNA mutations, including INDELS, donor DNA insertions, and large deletions.

42. We prefer to design three pairs of primer for genotyping; one primer pair amplifies within the gene of interest (∼1–2 kb for PCR amplification, the RNP targeting sequence should be included in this amplifying region), and the other two primer pairs amplifying the 5′ and 3′ regions of the targeted sequence (∼ 0.5 kb for PCR amplification, the reverse primer for 5′ upstream amplification and the forward primer for 3′ downstream amplification should locate closely (1–200 bp) to the start codon and stop codon of the gene of interest) (Figure 4).

**Figure 4:**
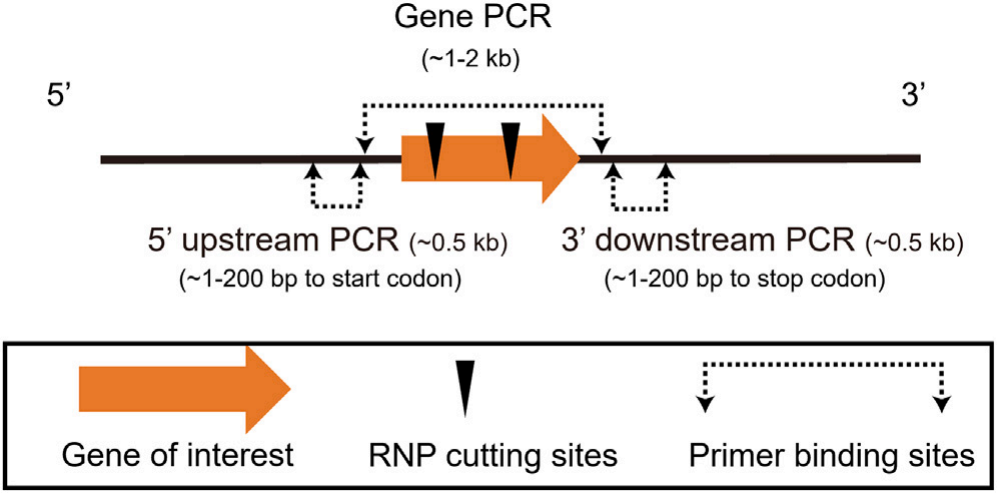
Schematic illustration of PCR primer pairs used for genotyping
43. Perform genotyping PCR followed the below program for the gene of interest. We used the *BUF1* gene as an example.

**Table:** 

Reagent	Amount
5× Q5 reaction buffer	5 μL
10mM dNTPs	0.5 μL
*BUF1* gene region forward primer (10 μM)	1.25 μL
*BUF1* gene region reverse primer (10 μM)	1.25 μL
Q5 High-Fidelity DNA Polymerase	0.25 μL
DNA extracted from quick extraction protocol	1 μL
Molecular water	15.75 μL
**Total**	**25 μL**

**Table:** PCR cycling conditions

Steps	Temperature	Time	Cycles
Initial Denaturation	98°C	30 s	1
Denaturation	98°C	10 s	30 cycles
Annealing	65°C	30 s	
Extension	72°C	2 min	
Final extension	72°C	2 min	1
Hold	4°C	2 min

***Note:*** The expected amplification size for wild-type is 1,648 bp, the size for mutants might vary based on the mutation pattern (Huang et al., 2021).

44. Perform genotyping PCR for 5′upstream, 3′ downstream of *BUF1* and *ACTIN* (loading control) with below program

**Table:** 

Reagent	Amount
10× standard Taq reaction buffer	2.5 μL
10mM dNTPs	0.5 μL
*BUF1* 5′ upstream or 3' downstream or *ACTIN* forward primer (10 μM)	0.5 μL
*BUF1* 5′ upstream or 3' downstream or *ACTIN* Reverse primer (10 μM)	0.5 μL
Taq DNA Polymerase	0.125 μL
DNA extracted from quick extraction protocol	1 μL
Molecular water	19.875 μL
**Total**	**25 μL**

**Table:** PCR cycling conditions

Steps	Temperature	Time	Cycles
Initial Denaturation	95°C	30 s	1
Denaturation	95°C	10 s	30 cycles
Annealing	See below note	30 s	
Extension	72°C	35 s	
Final extension	68°C	5 min	1
Hold	4°C	2 min

***Note:*** Annealing temperature for *BUF1* 5′ upstream or downstream is 54°C, while it requires 57°C for *ACTIN*. The expected amplification size for *BUF1* upstream, downstream and *ACTIN* is 311 bp, 458 bp and 503 bp, respectively.

## Expected outcomes

**Figure 5:**
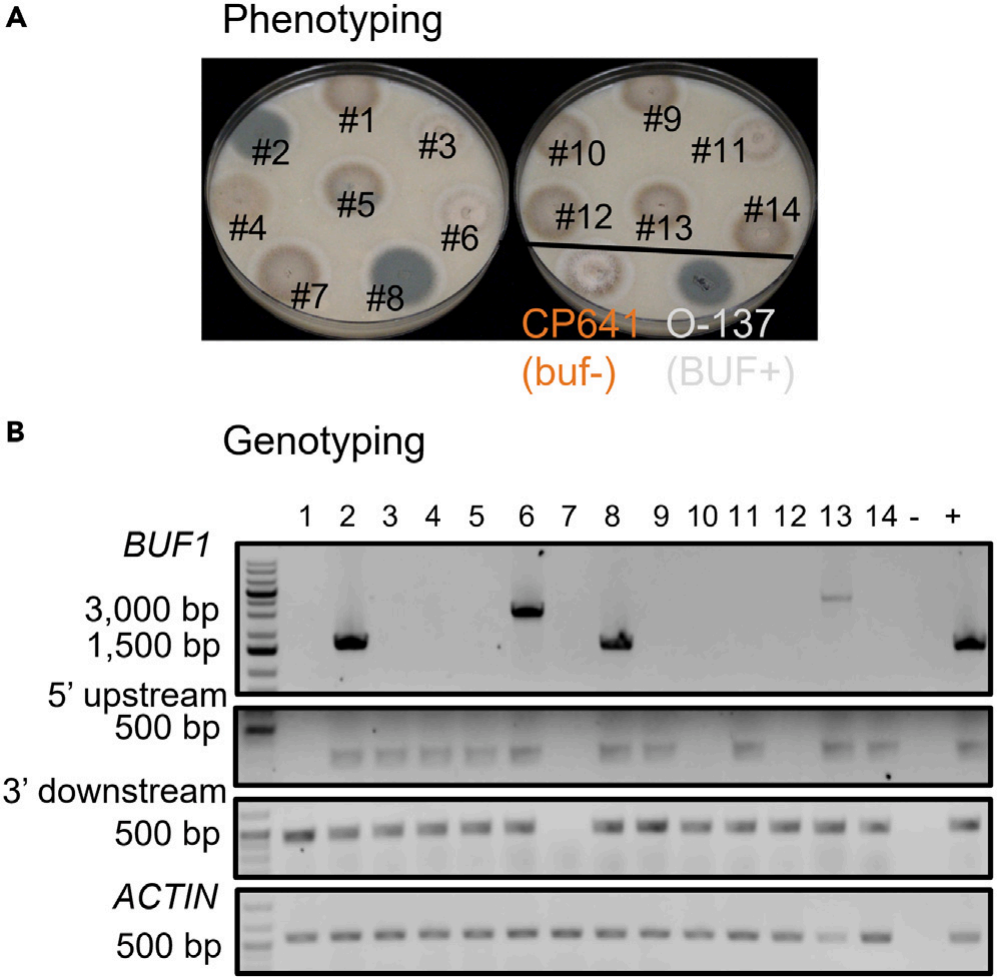
Representative phenotyping and genotyping outcomes at *BUF1* locus with *BUF1*-guide1 RNP and no-homology *HYG* DNA donor (A) Δ*buf1* showed the buff/orange mycelial color in OTA. CP641 (buf-) derived from O-137 is the positive strain for buff mycelia. O-137 (BUF+) is the wild-type strain used in this assay. (B) DNA extracted from the transformants in (A) were used for genotyping with *BUF1*, *BU1* 5′upstream, *BUF1* 3′ downstream and *Actin* primer pairs (loading control). – and + indicated the water (negative control) and O-137 genomic DNA (positive control) used in PCR reaction.

Gene disruption mutants can be generated by our method with an editing efficiency from ∼50% to 100% at *BUF1* locus (Figure 5A). Genotyping results suggested that large-scale insertion and deletions are common in the *buf1* mutants (Figure 5B). Please see more detailed analysis and discussion in the original manuscript (Huang et al., 2021).

## Limitations

Due to locus-dependent and physiological variables (e.g., gRNA efficiency, chromatin feature, sub-telomere/telomere location, and cell cycle), gene-editing efficiency can vary between loci and experiments. (Ceccaldi et al., 2016; Gisler et al., 2019; Huang et al., 2021; Schep et al., 2021; Xue and Greene, 2021). The use of two separate RNPs, targeting DNA regions within proximity to the desired location of editing may increase editing efficiency if single RNP transformation does not generate the desired mutation.

## Troubleshooting

### Problem 1

Cannot get the DNA template for T7 *in vitro* transcription from step 5.

### Potential solution

Double check the oligo concentration you used in your system, it requires 100 μM for oligos, which is 10× higher than the normal PCR reaction.

Confirm the complementary sequences shared between universal forward primer and target specific reverse primer.

### Problem 2

gRNA quality and concentration are low from step 6–7.

### Potential solution

A low yield of gRNA could result from the following:

Inefficient gRNA synthesis

Run DNA template before T7 *in vitro* transcription in 2% agarose gel to confirm the integrity of the initial DNA template. A single, strong signal near 66 bp is expected.

Exactly follow the T7 *in vitro* transcription step described in this protocol (step 6). Mix all the reaction components well by pipetting before starting the reaction. Extend the reaction time to 18 h for increased gRNA yield.

Inefficient gRNA purification

Follow the purification step described in Monarch® RNA Cleanup Kit (NEB Cat#T2050L), store the gRNA correctly prior to usage. For this, if we cannot immediately use the gRNA following purification, we store at −80°C for storage.

### Problem 3

Poor quality of protoplast from step 21.

### Potential solution

Avoid using the melanized mycelia for lysing enzyme digestion.

Prepare the lysing enzyme freshly.

Don’t over digest (> 4 h) the fungi.

Make sure you use miracloth rather than other filter materials in the filtering step after protoplasts are released.

Gently mix the protoplasts before hemocytometer quantification.

Keep released protoplast on the ice until you start the transformation.

### Problem 4

Buff phenotype is not visibly obvious from step 30.

### Potential solution

The buff phenotype is most visibly pronounced when *M. oryzae* is grown on OTA or RPA media. We found that plating the buff strains on CM produces less clear visible color differences when compared to the wild-type strain.

### Problem 5

Low gene editing efficiency from step 30.

### Potential solution

Many factors contribute to genome editing efficiency.

Guide design, if you are new for designing CRISPR gRNA, do an *in vitro* digestion assay followed the attached protocol (https://international.neb.com/protocols/2017/12/19/in-vitro-digestion-of-dna-with-engen-lba-cas12a-cpf1-neb-m0653), it will tell you whether your designed gRNA is compatible with Cas12a protein and the cutting efficiency.

Fungal protoplast, use freshly prepared protoplast for transformation. We found that freshly prepared protoplast with correct concentration always showed better transformation efficiency than frozen stored protoplast.

Temperature sensitivity, it has been shown before that LbCas12a is a temperature sensitive enzyme, with best editing efficiency at ∼28 or higher °C (Malzahn et al., 2019). Therefore, it is necessary to maintain your lab temperature around 28°C when you perform Cas12a transformation.

## Resource availability

### Lead contact

Further information and requests for resources and reagents should be directed to and will be fulfilled by the Lead Contact, David E. Cook, decook@ksu.edu.

### Materials availability

*M. oryzae* wild-type strains and derived mutants within this study are available upon reasonable request and may require permit.

## Data Availability

This study did not generate any unique datasets or code.

## Supplemental information

Table S1. Oligos used in this protocol, related to key resources table
